# Development of a common carp (*Cyprinus carpio*) pregnane X receptor (cPXR) transactivation reporter assay and its activation by azole fungicides and pharmaceutical chemicals

**DOI:** 10.1016/j.tiv.2017.02.023

**Published:** 2017-06

**Authors:** Anke Lange, Jenna Corcoran, Shinichi Miyagawa, Taisen Iguchi, Matthew J. Winter, Charles R. Tyler

**Affiliations:** a University of Exeter, Biosciences, College of Life & Environmental Sciences, Exeter EX4 4QD, United Kingdom; b Okazaki Institute for Integrative Bioscience, National Institute for Basic Biology, National Institutes of Natural Sciences, Department of Basic Biology, School of Life Science, SOKENDAI (Graduate University for Advanced Studies), 5-1 Higashiyama, Myodaiji, Okazaki, Aichi 444-8787, Japan; c AstraZeneca, Brixham Environmental Laboratory, Freshwater Quarry, Brixham, Devon TQ5 8BA, United Kingdom

**Keywords:** Pregnane X receptor, Common carp, Human, Transient transactivation assay, Pharmaceuticals, Azole fungicides

## Abstract

In mammals, the pregnane X receptor (PXR) is a transcription factor with a key role in regulating expression of several genes involved in drug biotransformation. PXR is present in fish and some genes known to be under its control can be up-regulated by mammalian PXR ligands. Despite this, direct involvement of PXR in drug biotransformation in fish has yet to be established. Here, the full length PXR sequence was cloned from carp (*Cyprinus carpio*) and used in a luciferase reporter assay to elucidate its role in xenobiotic metabolism in fish. A reporter assay for human PXR (hPXR) was also established to compare transactivation between human and carp (cPXR) isoforms. Rifampicin activated hPXR as expected, but not cPXR. Conversely, clotrimazole (CTZ) activated both isoforms and was more potent on cPXR, with an EC50 within the range of concentrations of CTZ measured in the aquatic environment. Responses to other azoles tested were similar between both isoforms. A range of pharmaceuticals tested either failed to activate, or were very weakly active, on the cPXR or hPXR. Overall, these results indicate that the cPXR may differ from the hPXR in its responses and/or sensitivity to induction by different environmental chemicals, with implications for risk assessment because of species differences.

## Highlights

• Establishment of a reporter gene assay for carp PXR.
• PXR-transactivation differs with agonists and species.
• Implications of species-differences in PXR for risk assessment.
• Fish might not be good models for studying potential chemical activation of human PXR.

## 1 Introduction

Aquatic species may be exposed to a wide range of xenobiotic compounds present in the environment, including human pharmaceuticals and personal care products. As a means to combat these exposures, many species have evolved an inducible system for xenobiotic metabolism that comprises of a number of enzymes including the cytochrome P450 superfamily (notably members of CYP1, 2, 3 and 4 subfamilies), conjugation enzymes (*e*.*g*. glutathione S transferases, UGTs, sulfotransferases), and transporter proteins such as P-glycoprotein and other multidrug resistance-associated proteins and organic anion transporter proteins. The ultimate purpose of this system is the detoxification and subsequent excretion of xenobiotic compounds from the exposed organism in order to minimise cellular toxicity.

Detoxification systems are conserved in vertebrates, and in mammals the pregnane X receptor (PXR; NR1I2) plays a major role. The PXR is an orphan nuclear receptor that has a pivotal role in transcriptional regulation of downstream detoxification pathways in humans and other mammalian models. CYP3A, an enzyme transcriptionally regulated by the PXR, is implicated in the metabolism of over 60% of pharmaceuticals in humans (Goodwin et al., 2002). The PXR has a large hydrophobic and flexible ligand binding domain (LBD, Kobayashi et al., 2004) and this enables its activation by a range of structurally diverse ligands. There are, however, species differences in the inducible nature of the PXR, even within the mammals, and this is likely to extend to other vertebrate groups. For example, whereas rifampicin (RIF) is a powerful activator of PXR in humans and rabbits, it fails to activate the rat or mouse PXR. Similarly, pregnenolone 16α-carbonitrile (PCN) activates the PXR in rodents, but shows a much reduced activity on human or rabbit PXR (Blumberg et al., 1998, Jones et al., 2000, Lehmann et al., 1998, Savas et al., 2000). The basis for this divergence is thought to be due to differences in the amino acid sequences in the ligand binding domain (Kliewer et al., 2002). As a result this variability in detoxification efficiency is likely to be reflected in the ability of different species groups to successfully process xenobiotics encountered in the environment.

The PXR pathway is well studied in mammals, but far less information is available on the role of this response pathway in fish. The PXR is present in some fish species, including zebrafish (*Danio rerio*), pufferfish (*Takifugu rubripes*), fathead minnow (*Pimephales promelas*) and medaka (*Oryzias latipes*), and it has been shown to be activated by some human receptor ligands (Bainy and Stegeman, 2004, Maglich et al., 2003, Milnes et al., 2008, Moore et al., 2000). The downstream effects of PXR activation in fish, however, are not well understood. We have previously shown that expression of a number of biotransformation genes (c*yp2k*, *cyp3a*, *gsta*, *gstp*, *mdr1* and *mrp2*) are up-regulated after exposure to RIF in carp primary hepatocytes (Corcoran et al., 2012) and exposure of carp to the mammalian PXR-agonist CTZ *in vivo* (Corcoran et al., 2014). Similarly expression of *pxr*, *cyp3a* and *mdr1* has been shown to be elevated following PCN exposure in zebrafish *in vivo* (Bresolin et al., 2005). It has also been demonstrated that RIF exposure results in increased CYP3A enzyme activity in primary hepatocytes from grass carp (*Ctenopharyngodon idellus*) and largemouth bass (*Micropterus salmoides*), (Li et al., 2008) and in a fathead minnow (FHM) cell line (Christen et al., 2010). However, direct involvement of the PXR in this response pathway in fish has yet to be established.

To better understand the role of the PXR in xenobiotic metabolism in teleosts, cDNA incorporating the full-length PXR coding region in carp was isolated, and the transactivation function of the PXR determined by establishing a carp PXR (cPXR) luciferase reporter assay expressing this receptor in transiently transfected cultured cells. *In vitro* nuclear receptor reporter assays have been established for various nuclear receptors, including oestrogen, androgen, thyroid and glucocorticoid receptors in fish (*e*.*g*. Bury et al., 2003, Lange et al., 2012, Oka et al., 2013, Todo et al., 1999) and applied to establish the roles of specific nuclear receptors in hormone signalling in fish. They have also been used as efficient chemical screening systems. Here we established a transactivation assay for the common carp PXR (cPXR) and investigated its activation by known mammalian PXR ligands RIF, dexamethasone (DEX) and clotrimazole (CTZ) and further compared responses of the cPXR with hPXR for a range of azole fungicides and pharmaceuticals present in the aquatic environment.

## 2 Materials and methods

### 2.1 Cloning of carp PXR sequence

Total RNA was isolated from frozen carp liver using Tri-reagent (Chomczynski, 1993) following the manufacturer's instructions. Following DNase treatment with RQ1 DNase (Promega, Southampton, UK), cDNA was synthesised from 1 μg total RNA using random hexamers and MMLV reverse transcriptase (Promega), according to the manufacturer's instructions. This cDNA was used as template for the polymerase chain reaction (PCR) amplification of a partial cPXR sequence using the degenerate oligonucleotides 5′-TYTTCAGRMGKGCSATGAAR-3′ and 5′-CCHGGVYGRTCTGGDGARAA-3′ designed in conserved regions from aligned PXR sequences in other, closely related species (zebrafish, grass carp, fathead minnow and rainbow trout). The partial cPXR sequence was amplified using GoTaq DNA polymerase (Promega) and the following PCR conditions: 96 °C for 2 min, followed by 35 cycles of denaturation at 94 °C for 1 min, annealing at 55 °C for 1 min and elongation at 72 °C for 3 min. A 865 bp product was obtained, purified using NucleoSpin Extract II columns (Macherey-Nagel, Dϋren, Germany) according to the manufacturer's instructions and sequenced (Eurofins Genomics, Ebersberg, Germany). BLAST searches (National Centre of Biotechnology Information (Altschul et al., 1990)) of the obtained sequences confirmed similarity with known PXR sequences.

The full cPXR sequence was obtained by rapid amplification of cDNA ends (RACE) using the SMARTer RACE cDNA Amplification Kit (Clontech, Mountain View, CA, USA) and gene specific primers Cc_GSP1: 5′-ACT ATG AAA GCT GGA GGA TGG GGA CGA G-3′ (antisense), Cc_GSP2: 5′-CTC ACT GCA CAT CAC AAG ACC TTC GAC A-3′ (sense) and Cc_GSP3: 5′-CCG CAA CCA GGA AAT AGT AGC ACT CAC C-3′ (sense) according to the manufacturer's instructions. Both 5′ and 3′ RACE products were purified as described previously, sequenced, and characterised using BLAST and Clustal W (Altschul et al., 1990, Larkin et al., 2007). A neighbour-joining phylogenetic tree was constructed in MEGA7 (Kumar et al., 2016) using a 1000 replicate bootstrap analysis.

### 2.2 cPXR expression plasmid

A full-length cPXR fragment was amplified using the primers Cc_PXR_F_K_*Bam*HI: 5′-GCG GAT CCG CCA CCA TGT GCT TGC TTC AGC TCA GG-3′ and Cc_PXR_R_EcoRV: 5′-CCG ATA TCG TCC TCG CTG GTT TTG ACT G-3′ designed at the extreme ends of the obtained 5′- and 3′-RACE sequences, incorporating restriction sites (*Bam*HI and *Eco*RV) as well as the kozac sequence (which enhances ribosomal binding at the start codon during transcription) in the case of the 5′ primer. For this, cDNA was reverse transcribed from 1 μg of carp liver total RNA as described above and amplified using the Advantage 2 DNA polymerase mix (Clontech) according to the manufacturer's instructions. The resulting PCR product was purified and sequenced to confirm the full sequence length and sub-cloned into pGEM-T Easy vector (Promega) following the manufacturer's recommendations. Plasmid DNA was isolated using the Wizard Plus SV minipreps DNA Purification system (Promega) according to manufacturer's instructions. Using the *Bam*HI and *Eco*RV sites, the cPXR sequences was subsequently ligated into the eukaryotic expression vector pcDNA3.1(+) (Invitrogen) using the correspondent restriction enzymes (New England Biolabs, Hitchin, UK) and T4 DNA ligase (Promega).

### 2.3 hPXR expression plasmid

Human PXR (hPXR) clone cDNA was purchased from Promega. 1 ng cDNA was served as template to amplify the full hPXR sequence using PrimeStar Max DNA polymerase (Takara, Ohtsu, Japan) and the primers hPXR_F_*Bam*HI: 5′-GGA TCC GCC ATG ACA GTC ACC AGG ACT C-3′ and hPXR_R_*Xba*I: 5′-TCT AGA TCA GCT ACC TGT GAT ACC GAA CAA-3′. The following PCR protocol was employed: 21 cycles of denaturation at 98 °C for 10 s, annealing at 60 °C for 5 s and elongation at 72 °C for 15 s. The purified product was then ligated into the eukaryotic expression vector pcDNA3.1(+) (Invitrogen) using the correspondent restriction enzymes and T4 DNA ligase (Promega).

### 2.4 Construction of pGL4.24-6xPXRE -reporter plasmid

**Fig. 1:**
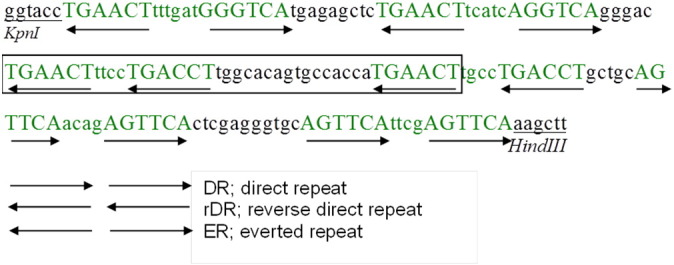
Sequence of the 6xPXRE reporter construct. Nucleotides shown in green are PXRE sequences. Capital letters mark the conserved motifs. The boxed section is a DR4 based on that published by Xie et al. (2000). This sequence was inserted into the pGL4.24 vector using *Kpn*I and *Hind*III restriction sites (underlined) to create the PXRE reporter vector. Arrows indicate the direction of the nuclear response element repeat motif.

A reporter vector was constructed based on several variations of the PXR response element (PXRE; Fig. 1). The PXRE occurs either as a direct or everted repeat of the consensus motif AGTTCA, spaced by between 3 and 8 nucleotides. The PXRE sequence created here was based on that reported in Xie et al. (2000). Initially, two complementary long chain oligos 5′-TGA GAG CTC TGA ACT TCA TCA AGG TCA GGG ACT GAA CTT TCC TGA CCT TGG CAC AGT GCC ACC ATG AAC TTG CCT GAC CTG CTG CAG TTC AAC AGA GTT CAC TCG AGG GT-3′ and 5′-ACC CTC GAG TGA ACT CTG TTG AAC TGC AGC AGG TCA GGC AAG TTC ATG GTG GCA CTG TGC CAA GGT CAG GAA AGT TCA GTC CCT GAC CTT GAT GAA GTT CAG AGC TCT CA-3′ were annealed to create double stranded DNA, containing four PXRE motifs. To maximise reporter efficiency, a further PXRE motif was added either side of the previously generated long chain oligo using PCR. One nanogram of the annealed DNA served as template and was amplified using the primers PXRE_*Kpn*I 5′-TGA GGT ACC TGA ACT TTT GAT GGG TCA TGA GAG CTC TGA ACT TCA TCA AGG-3′ and PXRE2_*Hind*III 5′-CCT AAG CTT TGA ACT CGA ATG AAC TGC ACC CTC GAG TGA ACT CTG TTG-3′ each containing one restriction enzyme site (*Kpn*I and *Hind*III, respectively) and PrimeStar Max DNA polymerase (Takara). The following PCR conditions were employed: 21 cycles of denaturation at 98 °C for 10 s, annealing at 60 °C for 5 s and elongation at 72 °C for 30 s. After purification, and using the appropriate restriction enzymes *Kpn*I and *Hind*III, the obtained fragment was subsequently ligated into the pGL4.24[*luc2P*/minP] vector (Promega) which contains the firefly (*Photinus pyralis*) luciferase (*luc2P*) gene, to create the pGL4.24-6xPXRE reporter construct.

### 2.5 Transactivation assays

COS-7 cells (ATCC CRL-1651) were cultured in phenol-red free Dulbecco's modified Eagle's medium containing 1000 mg/L glucose (Life Technologies Ltd, Paisley, UK), supplemented with 2 mM l-glutamine (Life Technologies Ltd) and 10% charcoal/dextran treated foetal bovine serum (FBS; Hyclone, South Logan, UT, USA). The cells were maintained at subconfluent densities and sub-cultured when reaching 80% confluency.

For transfection assays, cells were seeded in 24-well plates at 5 × 10^4^ cells well^− 1^ in phenol-red free DMEM (supplemented with 10% charcoal/dextran-treated foetal bovine serum (Hyclone). After 24 h, the cells were transiently transfected with 200 ng of either pcDNA3.1(+)/cPXR or pcDNA3.1(+)/hPXR, 400 ng of reporter construct (either MMTV/luc2/pGL4 (Promega) or pGL4.24-6xPXRE) and 100 ng of pRL-TK (driving the *Renilla reniformis* luciferase gene as an internal control to normalise for variations in transfection efficiency) using Fugene HD transfection reagent (Promega) in serum-free medium according to the manufacturer's protocol. Four hours after transfection, cells were treated with PXR agonists (see below). Forty-eight hours after transfection, cells were lysed and the luciferase activities of the cells were measured by a chemiluminescence assay with Dual-Luciferase Reporter Assay System (Promega) according to the manufacturer's instructions. Luminescence was measured using an Infinite 200 Pro plate reader (Tecan, Grödig, Austria). Promoter activity was calculated as firefly (*Photinus pyralis*)-luciferase activity/sea pansy (*R. reniformis*)-luciferase activity. All transfections were done at least three times, employing triplicate sample points in each experiment.

### 2.6 Chemical screening

Initially, transcriptional assays were used to measure activation of both cPXR and hPXR by the prototypical human PXR ligands RIF and DEX at a concentration of 10^− 6^ M, comparing two separate reporter constructs in assessing for PXRE function. In addition to the pGL4.24-6xPXRE reporter construct, a commercially available MMTV/luc2/pGL4 reporter vector (Promega) was tested as a reporter plasmid (*i*.*e*. for PXRE function), as it is known to contain various nuclear receptor response elements in the promoter region.

The pGL4.24-6xPXRE construct was subsequently used as the reporter construct in transcriptional assays to establish concentration-response curves for the receptor agonists RIF and CTZ in the concentration ranges of 10^− 11^ M to 10^− 5^ M for both cPXR and hPXR. Subsequently, pharmaceuticals of different therapeutic classes were screened for their ability to activate hPXR and cPXR. These were: the non-steroidal anti-inflammatory drugs (NSAIDs) diclofenac (DIC), ibuprofen (IBU) and ketoprofen (KTP); the fibrates clofibric acid (CFA) and gemfibrozil (GEM); the β–blockers propranolol (PRP) and atenolol (ATN); the (anti)oestrogens 17α-ethinyloestradiol (EE2) and tamoxifen (TAM); and the azole antifungal drugs ketoconazole (KTZ) and miconazole (MCZ). Propiconazole (PCZ), an agricultural azole antifungal was also included in the analyses. For all compounds, dose-response curves were established in the concentration ranges of 10^− 11^ M to 10^− 5^ M, for both cPXR and hPXR.

All compounds (obtained from Sigma-Aldrich) were dissolved and diluted in DMSO and added to the medium. The final solvent concentration in the transactivation assays was 0.1% DMSO and control wells were dosed with 0.1% DMSO only.

### 2.7 Data analysis

Data are presented throughout as mean ± standard error of the mean (SEM). All transfections were performed in triplicate and repeated three times on cells with different passage numbers. Concentration–response data using a four-parametric curve fitting and EC50 (for agonists) were analysed using GraphPad Prism (Graph Pad Software Inc.). Chemical responses were normalised against their relevant respective controls. Statistical analyses were carried out using SigmaPlot® software (Systat Software, Inc.) and *p* < 0.05 was considered statistically significant.

## 3 Results

### 3.1 Cloning of full-length cPXR and sequence analysis

**Fig. 2:**
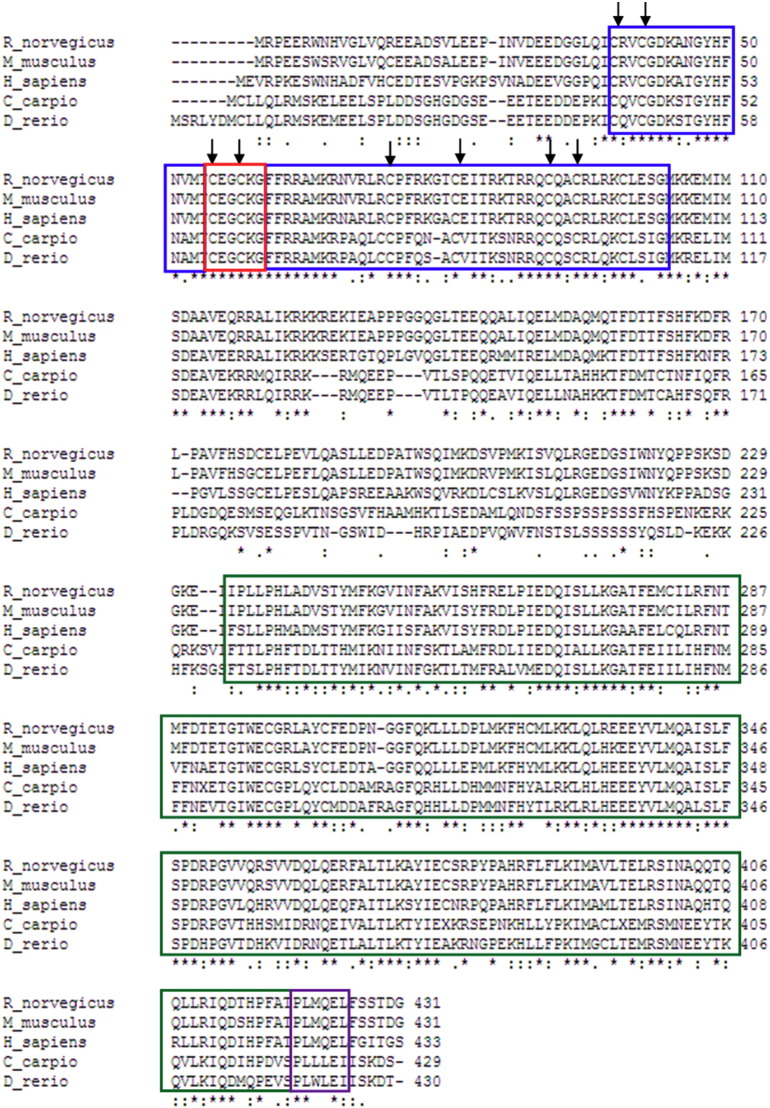
cPXR amino acid sequence aligned with PXR sequences of other animal species. The highly conserved DBD (blue outline) consists of two C4-type zinc fingers and includes a P-box motif (red outline). The conserved LBD (green outline) includes the AF-2 motif (purple outline). Accession numbers of sequences used for alignment: AAH17304 (*H. sapiens*); NP_443212 (*R. norvegicus*); NP_035066 (*M. musculus*); NP_001092087 (*D. rerio*).

**Fig. 3:**
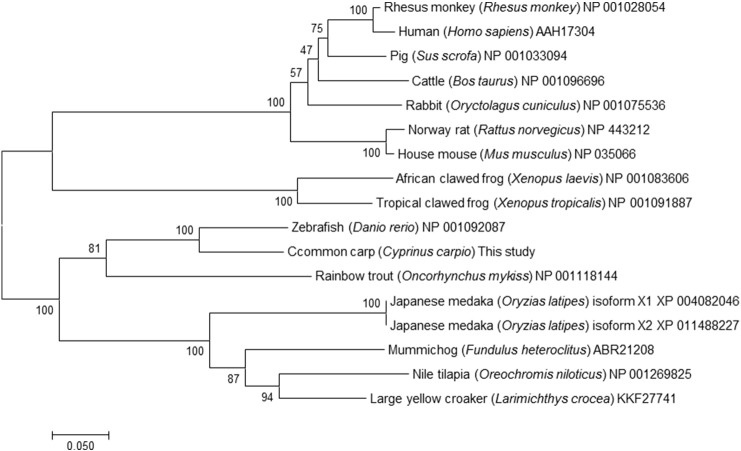
Evolutionary relationships of PXR. The neighbour-joining phylogenetic tree was constructed based on full length amino acid sequences. The scale bar represents 0.05 substitutions per site.

Using 5′- and 3′-RACE PCR, the full-length cDNA sequence of cPXR was isolated from carp liver. The fragment obtained was composed of 1696 nucleotides, containing 262 and 99 base pairs of the 5′- and 3′-untranslated regions, respectively. The open reading frame of the cPXR nucleotide sequence predicts a protein of 444 amino acid residues. The full-length sequence obtained for cPXR has been deposited in the GenBank database (accession # KX241860). Sequence analyses showed that cPXR has a highly conserved DNA binding domain (DBD) consisting of two C4-type zinc fingers and including a P-box motif (CEGCKG; a sequence essential for DNA-binding specificity) and a conserved ligand-binding domain (LBD) including the AF-2 motif (PLxxEx), essential for co-regulator interaction during ligand binding of transcription factors. BLASTp analysis (Altschul et al., 1997) confirmed sequence identity and revealed a high homology between the amino sequence of the DBD of the cPXR with the zebrafish PXR (98%) and human PXR (74%) (Fig. 2). The overall amino acid identity of cPXR with other PXR sequences was between 46 and 78%. In a phylogenetic analysis, the teleost PXR forms a distinct clade with the cPXR most similar to PXR of zebrafish (Fig. 3).

### 3.2 Comparison of reporter plasmids

**Fig. 4:**
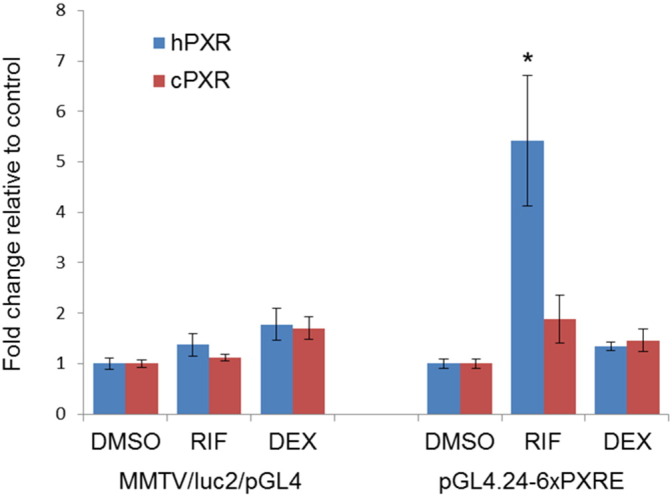
Activation of cPXR and hPXR by rifampicin (RIF) and dexamethasone (DEX) mediated by two different luciferase reporter vectors. An asterisk denotes the treatment is significantly different from the corresponding DMSO control group (*p* < 0.05).

Initially, both cPXR and hPXR reporter assays were investigated using two different reporter vectors, namely the pGL4.24-6xPXRE construct and the commercial MMTV/luc2/pGL4 plasmid, to test for responses to PXR activation (*i*.*e*. for a functional PXRE) (Fig. 4). The test system transfected with pGL4.24-6xPXRE showed significant activation of the hPXR in those cells exposed to 1 μM RIF compared with the control. cPXR activity also appeared to be higher in cells exposed to 1 μM RIF compared with control cells, although this was not supported statistically (*p* > 0.05). The test system transfected with the MMTV/luc2/pGL4 plasmid in contrast showed no significant treatment-related activation with respect to either hPXR or cPXR. pGL4.24-6xPXRE was used as the reporter plasmid in all subsequent transactivation concentration-response assays.

### 3.3 Responses of PXRs to known PXR agonists

**Fig. 5:**
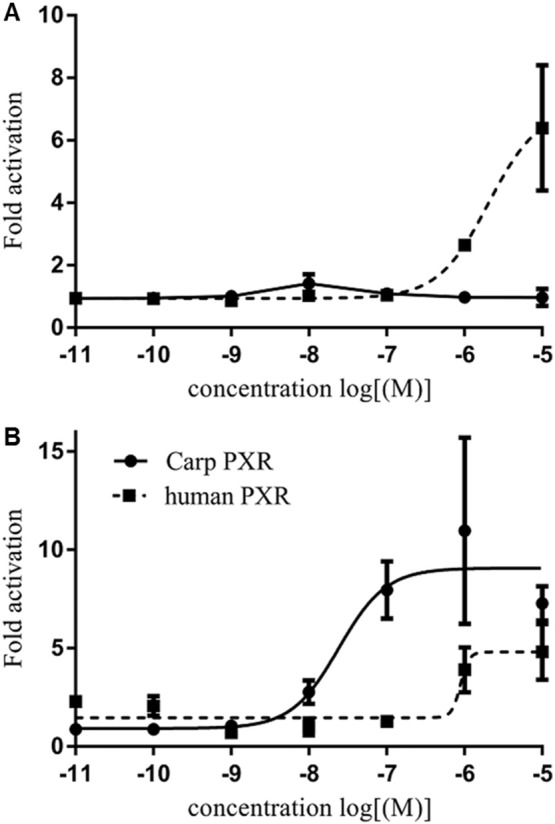
Concentration-response curves for carp (solid line) and human (dashed line) PXR activation on exposure to (A) rifampicin and (B) clotrimazole for 44 h at concentration between 10 μM and 10 pM (10^− 5^ and 10^− 11^ M). Data are presented as x-fold activation relative to DMSO control.

Concentration-dependent transactivation of hPXR was observed after exposure to concentrations between 10 μM and 10 pM RIF (Fig. 5). RIF activated hPXR with a maximal effect (*E*_max_) 6.4 times that of the control and an EC_50_ of 2.04 μM. cPXR was not activated by RIF.

CTZ activated both the hPXR and cPXR, but with different potencies. For hPXR, the EC_50_ was 0.88 μM with an *E*_max_ of 4.8, and for cPXR, the EC_50_ was 0.024 μM with an *E*_max_ of 10.9.

### 3.4 Activation of cPXR and hPXR by pharmaceuticals and fungicides

**Fig. 6:**
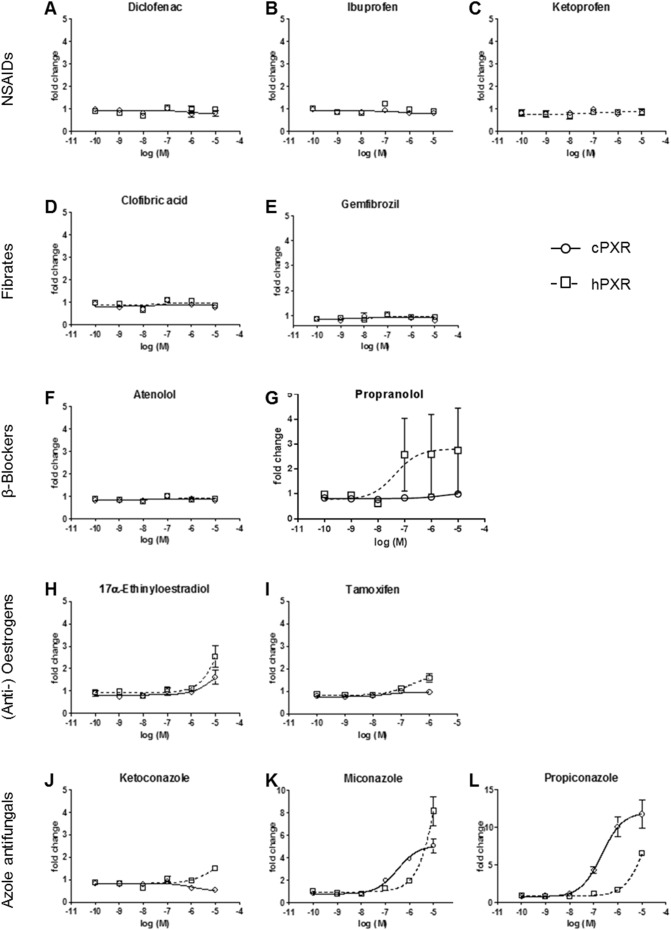
Concentration–response profiles of cPXR and hPXR exposure to: the NSAIDs diclofenac (A), ibuprofen (B) and ketoprofen (C); the fibrates clofibric acid (D) and gemfibrozil (E); the β-blockers atenolol (F) and propranolol (G); the (anti-)oestrogens 17α-ethinyloestradiol (H) and tamoxifen (I); and the antifungals ketoconazole (J), miconazole (H) and propiconazole (L). Results are expressed as mean change normalised against their relevant respective controls ± SEM. Dose-response-curves were fitted by non-linear regression.

All three NSAIDs (DIC, IBU and KTP), both fibrates (CFA and GEM) and the β-blocker (ATN) did not induce transactivation of either cPXR or hPXR at any of the concentrations tested (Fig. 6A–F). Propranolol activated hPXR (EC_50_ = 44 nM), but had no effect on cPXR (Fig. 6G). EE2 appeared to induce luciferase activity in both cPXR and hPXR, but did so only at high (10 μM) concentrations (Fig. 6H). TAM had no effect on cPXR and only induced transactivation of hPXR at 10 μM (Fig. 6I). This treatment level, however, would likely have been approaching a toxicity level; 100 μM TAM was toxic to COS-7 cells in both, cPXR and hPXR assays as established by an inhibited cell growth. The azole antifungals MCZ and PCZ induced transactivational activity of both, cPXR and hPXR. For cPXR, the estimated EC_50_s were 0.315 μM MCZ and 0.213 μM PCZ. For hPXR, higher concentrations of MCZ and PCZ were required to induce transactivation of cPXR and hPXA and full dose-response curves were not obtained (Fig. 6K, L). KTZ had no effect on cPXR or hPXR transactivation (Fig. 6J).

## 4 Discussion

In this study, we cloned a full-length PXR sequence from the common carp which, based on sequence analyses, was identified as an orthologue of human and mouse PXR. The percent identities of the cPXR DBD and LBD reported here are in agreement with those published for other fish species, including the zebrafish, another cyprinid species (Bainy et al., 2013, Krasowski et al., 2005, Milnes et al., 2008). Of all of the nuclear receptors, vertebrate PXR sequences show the greatest sequence and functional differences between species. The amino acid sequence of the PXR LBD shows an unusually high sequence divergence between different orthologues (Handschin and Meyer, 2003, Iyer et al., 2006). It is speculated that the varying degrees of sequence conservation of the vertebrate PXR LBD and resulting broad ligand specificity of PXR might reflect variation in exogenous PXR ligands as well as species physiology (Krasowski et al., 2011).

Here, using the pGL4.24-6xPXRE construct as reporter plasmid, the hPXR was activated by RIF and by CTZ, both well-established PXR agonists in humans. The hPXR was activated by RIF with an EC_50_ of 2.04 μM, and *E*_max_ of 6.4 μM, and CTZ with an EC_50_ of 0.88 μM, both of which are comparable to those previously reported in humans (Bertilsson et al., 1998, Blumberg et al., 1998, Lemaire et al., 2004, Milnes et al., 2008, Moore et al., 2000, Sinz et al., 2006, Svecova et al., 2008). DEX, conversely, failed to activate the hPXR. Although DEX is a potent agonist of the rodent PXR, it is only a weak agonist of hPXR (Kliewer et al., 2002) and the response seen here is comparable with that reported previously for the hPXR following treatment with 2 μM DEX (Luo et al., 2002). In summary, the activation of the hPXR in the transfection assay, established for RIF, CTZ and DEX, were consistent with previous reports on the responsiveness of the hPXR to these compounds.

In this study, cPXR did not respond to RIF, supporting findings for the zebrafish PXR (Moore et al., 2000). Contrasting with these cyprinid fish RIF (50 μM) has been shown to activate PXR from the pufferfish (*Takifugu rubripes*) (Milnes et al., 2008). Interestingly, RIF also shows species differences in mammals as a PXR ligand. For example, RIF activates PXR strongly in humans and rabbits, but does so only relatively weakly in rodents (Kliewer et al., 2002). This difference is thought to be due to sequence differences in the LBD. This may also be the case for fish; carp and zebrafish PXR share high sequence similarity (79%) whereas Fugu Sp PXR shares only 52% and 51% sequence homology with carp and zebrafish PXR, respectively. In mammals, this species-specificity for PXR ligand activation parallels responses (or lack thereof) in expression of *cyp3a* to the same ligands (Kliewer et al., 2002, Luo et al., 2002); *cyp3a* is transcriptionally regulated directly by PXR. The lack of a response of the cPXR to RIF seen here is, however, contrasts with previous gene expression profiles for *cyp3a* (Corcoran et al., 2012). In carp, we have shown previously that *cyp3* and a number of genes associated with the PXR in mammals were up-regulated in a concentration-dependent manner by RIF, and in some cases at concentrations as low as 0.1 μM. Furthermore, this induction was inhibited by co-exposure to ketoconazole, which is known to antagonise the interaction of RIF with the PXR. In other fish species too, RIF has been shown to induce CYP3A enzyme activity *in vitro*. Examples include in FHM cell line (Christen et al., 2010), and in primary hepatocytes from grass carp and largemouth bass, albeit at relatively high exposure levels (highest net increase in aminopyrine *N*-demethylase activity was reached at 52.43 and 45.28 μM, respectively (Li et al., 2008)). The lack of response of the cPXR to RIF in the present study is, therefore, somewhat unexpected, and may suggest that the observed up-regulation of teleost biotransformation genes and enzymes by RIF may involve non-PXR mediated mechanism(s).

In mammals, there is evidence for considerable crosstalk between PXR and other nuclear receptors and transcription factors, including farnesoid X receptor (FXR), glucocorticoid receptor (GR) and vitamin D receptor (VDR), in the regulation of biotransformation genes (Pascussi et al., 2008). This sort of interaction may account for the discrepancy between the responses of teleost CYP3A at the gene and enzyme level previously observed and the PXR activation profile presented here. For example, in rodents GR has also been shown to regulate expression of *cyp3a*, possibly explaining the inconsistencies between induction of *cyp3a* expression against a weak activation of the PXR by DEX (a GR ligand) (Quattrochi and Guzelian, 2001).

It is also the case that PXR activation may require various co-factors, co-repressors, transcription factors and/or complexes that interact with the PXR or the response element (Quattrochi and Guzelian, 2001), as is common for other nuclear receptors. Whether all of the required factors for the activation of cPXR by RIF are present in the transfection assay cell systems is not known.

The cPXR was more sensitive to CTZ compared with the human isoform and this is consistent with findings in other fish species where PXR has been shown to be highly responsive to CTZ. Examples include the zebrafish PXR shown to be up regulated in transfection assays by 8-fold (above controls) for exposure concentrations between 0.5 and 50 μM (Bainy et al., 2013, Milnes et al., 2008, Moore et al., 2000) and in the FHM PXR by up to 35-times for exposure to 50 μM CTZ (Milnes et al., 2008).

CTZ concentrations in effluent and surface waters are generally in the low ng l^− 1^ range, but in sewage effluent have been reported at concentrations up to 1.8 μg l^− 1^ (Peng et al., 2012) which are approaching the lowest effect concentration reported here. Moreover, CTZ has been shown to bio-concentrate in fish (Corcoran et al., 2014), with a plasma bioconcentration factor (BCF_plasma_) of between 20 and 45, leading to the possibility of higher target tissue concentrations of this compound in exposed fish.

The PXR activation by CTZ demonstrated in fish does not always parallel expression of some PXR-associated genes, in particular *cyp3a*, either *in vivo* or *in vitro* (Bresolin et al., 2005, Corcoran et al., 2012, Crago and Klaper, 2011, Wassmur et al., 2013). We have though previously demonstrated up-regulation in the expression of a number of biotransformation genes, (including *cyp3a*) in carp exposed *in vivo* for 10 days to measured water concentrations of 17 μg CTZ l^− 1^ (Corcoran et al., 2014). Some inconsistencies between PXR activation and gene responses in the different carp studies, however, are still apparent. In this study cPXR was activated *in vitro* by CTZ at concentrations 100 times lower than those shown to induce expression of biotransformation genes *in vivo*. It is possible that a certain threshold level of PXR activation is required to subsequently induce activation of downstream gene targets. Equally, it is possible that the PXRE construct does not reflect the sensitivity of carp PXRE motifs in target genes *in vivo*. An alternative explanation is that activation of the teleost PXR does not lead to induction of biotransformation genes, as is known to occur in mammals.

Globally, pharmaceuticals have been detected in surface waters and wastewater treatment work effluents (reviewed in Aus der Beek et al., 2016) and are of concern to receiving biota due to their biological specificity and potency (reviewed in Corcoran et al., 2010). In mammals, PXR is known to play a significant role in regulating drug biotransformation (Liddle and Goodwin, 2002) whereas in fish, very limited information is available on the involvement of PXR in drug biotransformation. Various pharmaceuticals have been described as CYP3A inducers in human through their ability to activate PXR (Sinz et al., 2006). Previous studies have also indicated a potential involvement of PXR in regulating selected genes involved in drug metabolism in fish (Corcoran et al., 2012). Five out of 11 pharmaceuticals we tested (PRP, EE2, TAM, KTZ and MCZ) were found to activate hPXR, albeit only partially and some were very weak in their capacity to do so. cPXR, on the other hand, was activated by EE2 and MCZ. The lack of hPXR transactivation by IBU is consistent with previous findings, but for DIC contrasts with that reported previously for the hPXR (Creusot et al., 2010, Sinz et al., 2006). Contrasting with our findings here that IBU and CFA had no effect on cPXR transactivation, previously using carp hepatocyte cultures, we found that these compounds induce an up-regulation in expression of the biotransformation genes c*yp2k*, *cyp3a*, *gsta*, *gstp*, *mdr1* and *mrp2* (Corcoran et al., 2012) and for CFA this was mirrored for an *in vivo* exposure in carp (Corcoran et al., 2015). The lack of hPXR activation by the fibrates (GEM and CFA) is consistent with a previous finding for hPXR in transactivation assays (Sinz et al., 2006). For some compounds, in particular CTZ and PRP, a higher variability was observed in response to the higher agonist concentrations tested and onset of cytotoxicity or solubility are both possible explanations for this variability. Cytotoxicity is an unlikely factor as the cells did not show any signs of morphological changes. In terms of solubility, of the compounds yielding variable responses, only the highest CTZ concentration was bordering water solubility limits. The reason for high variability for some a few of the data points across multiple experiments appears to relate to the variation in the technical replicates, which we are unable to account for.

The β-blocker PRP activated the hPXR, but neither of the β-blockers tested activated the cPXR transactivation assay. For both β-blockers tested, no detectable transactivation of hPXR has been demonstrated previously (Sinz et al., 2006). We have though shown an up-regulation of c*yp2k*, *cyp3a*, *mdr1* and *mrp2* gene expression in carp primary hepatocytes in response to PRP exposure previously (Corcoran et al., 2012).

EE2 induced transactivation of both cPXR and hPXR albeit only at the highest exposure concentration tested (10 μM), whereas TAM induced transactivation of hPXR only, at 1 μM and did so only weakly. Transactivational activity at the hPXR has been shown previously for both drugs (Sinz et al., 2006).

The azole fungicides were the only class of compounds showing consistent effects between both hPXR and cPXR. In mammals, azoles are known to be both inhibitors and inducers of specific hepatic biotransformation systems, the effects being mediated through PXR (Hester et al., 2012, Huang et al., 2007, Sun et al., 2005, Wang et al., 2007). Both MCZ and PCZ induced transactivation of cPXR and hPXR and they were more potent in the cPXR. *In vivo* studies have shown PCZ induces CYP2B and CYP3A isoforms in rat and mouse liver as well as hepatic *cyp3a* gene expression in fathead minnow (Skolness et al., 2013, Sun et al., 2005). Collectively, these findings indicate that PCZ is a potent activator of PXR and suggests a common pathway between fish and mammals. In line with our findings, MCZ has been identified in humans as potent PXR ligand, also inducing CYP3A4 (Dvorak, 2011, Svecova et al., 2008). To the best of our knowledge, no other data are available on the effects of MCZ on fish. In our *in vitro* assays, KTZ did not induce transactivation of cPXR or hPXR. In carp hepatocytes, co-exposure to RIF and KTZ resulted in an inhibition of the gene expression responses of *cyp2k* and *cyp3a*, and KTZ alone had an effect on *cyp2k*, but no effect on *cyp3a* (Corcoran et al., 2012). In rainbow trout and killifish, KTZ is known to induce CYP3A protein expression, but inhibit its catalytic activity *in vivo* and *in vitro* (Hegelund et al., 2004).

Overall, our data show species differences in the potency of xenobiotics to transactivate PXR *in vitro*, however, in all cases cPXR transactivation occurred at concentrations exceeding those with environmental relevance. As an example, the EC50s determined for the most potent cPXR agonists were 0.315 μM MCZ and 0.213 μM PCZ, whereas measured average environmental concentrations are generally in pico- to nanomolar ranges (*e*.*g*. 0.019 nM MCZ and 0.85 nM PCZ) (Battaglin et al., 2011, Roberts and Bersuder, 2006). Similarly for EE2, low micromolar concentrations were required to induce a weak transactivation of cPXR and the average concentration of EE2 in surface waters globally is sub-nanomolar (Aus der Beek et al., 2016). We are not excluding the possibility that environmental exposure to these compounds does not pose any possible risk for adverse effects, but the likelihood is low for any individual compound. There are, however, complex mixtures of PXR receptor agonists in the aquatic environment and when in combination, compounds with low individual efficacy can activate hPXR in a synergistic manner (Delfosse et al., 2015).

There are considerable challenges when comparing data sets for PXR activation across the different published studies even for the reporter gene assay systems alone. The reasons for this include that a variety of different cell lines have been used, and that some transactivation assays employ a reporter construct consisting only of the PXR LBD rather than the full PXR sequence. To illustrate the differences in transactivation assays that can occur for sequence inclusions of different sizes, exposure to CTZ was shown to result in a considerably lower (90% lower) activation of a zebrafish full-length PXR compared with a zebrafish PXR-LBD construct (Bainy et al., 2013). This serves to emphasise that while reporter assays offer excellent *in vitro* tools to screen for PXR activation, functional studies either employing hepatocyte cultures or *in vivo* experimental approaches are needed for the validation of any effects seen.

Overall, the data presented suggest that the reporter gene assay developed for activation of cPXR is a sensitive and reproducible model system for characterising the ligand activation profile of the PXR in carp. This assay can be applied effectively for screening chemicals, including pharmaceutical, and/or environmental samples for PXR activation, for use in prioritising risk assessments. Application of the assay has identified possible differences between the PXR associated xenobiotic-metabolising pathway in mammals compared with in fish. This being the case fish (carp) may be poor models for studying chemical activation of human PXR function. Our findings further indicate that fish may have different metabolic pathways for detoxification of some of the key chemicals of environmental concern with possible implications for hazard identification and risk assessment.

## Transparency document

Transparency document Image 1
